# Journée Scientifique Dématérialisée de la SFMTSI (Ancienne Société de Pathologie Exotique) Pilotée à Partir de ses Bureaux sis au Pavillon Laveran, Hôpital de la Salpêtrière, Paris Jeudi 20 Mai 2021

**DOI:** 10.48327/mtsibulletin.n1.2021.92

**Published:** 2021-04-20

**Authors:** A. Epelboin, P. Gazin, C. Goujon, É. Pichard, S. Le Gurun

**Affiliations:** SFMTSI. Hôpital Pitié-Salpêtrière - Pavillon Laveran, 47-83 Boulevard de l'Hôpital, 75651 Paris cedex 13

Cette journée a eu pour but d'inciter les praticiens, les chercheurs, les décideurs publics et privés, économiques et politiques, à considérer et à mieux anticiper l'impact sous les tropiques des pollutions environnementales rurales et urbaines sur la santé humaine, animale, végétale, marine, et microbiologique: les conséquences de l'exposition à ces substances toxiques sont particulièrement néfastes quand elle est associée à des complexes pathogènes spécifiques des tropiques, à la pauvreté et au sous-développement.

Il ne s'agit pas de dresser un catalogue de toutes les pollutions environnementales, mais de situer chaque polluant présenté dans une perspective écosystémique, pluridisciplinaire de santé publique et aussi individuelle (Une seule santé/*One Health*), en tenant compte des histoires, ressentis et représentations locales. Malgré leur importance, des sujets majeurs, comme les devenirs des matières plastiques non carbonisées, les perturbateurs endocriniens, ou les pollutions radioactives, n'ont pas été traités faute de temps.

Nous avons traité des conséquences des pollutions aériennes urbaines par les fumées de combustion des ordures, des moteurs, des foyers domestiques…, à partir de l'exemple d'écosystèmes urbains togolais et ivoiriens; il a aussi été question des impacts des pollutions urbaines et agricoles sur la prolifération des moustiques, de dysplasies faciales chez des chimpanzés sauvages attribuées aux produits phytosanitaires utilisés dans les champs qu'ils ravagent, et de la chlordécone aux Antilles françaises; ont été abordés également les impacts sur la santé des populations vivant à proximité d'exploitations minières et pétrolières, industrielles et artisanales et plus particulièrement des pollutions par les métaux lourds (Hg, Pb…), associées aux exploitations aurifères.

À côté des risques sanitaires proprement dits, les répercussions sociales, politiques, économiques, à l'échelon local, national et international ont été évoquées, notamment au cours de trois tables rondes d'une trentaine de minutes organisées entre les intervenants de chaque session (« pollutions aériennes », « pollutions par pesticides et engrais », « pollutions minières et pétrolières ») et alimentées par les questions et réflexions écrites des participants.

## Programme

**9 h Introduction par les organisateurs**

**9 h 10 Pollutions aériennes**

**9h10-9h30** Émission anthropiques, qualité de l'air urbain et impact sanitaire en Afrique sub-saharienne. Cathy Liousse, physico-chimiste, CNRS, Laboratoire d'aérologie de Toulouse

**9h30-9h50** Affections broncho-pulmonaires et pollutions urbaines. Eric Pichard, médecin, infectiologue, Institut Pasteur, APHP, SFMTSI

**9h50-10h20** Discussion

**10h20-10h30** Pause

**10h30 Pollutions par pesticides et engrais**

**10h30-10h50** Les chimpanzés sauvages, victimes et sentinelles des pollutions environnementales? Sabrina Krief, primatologue, UMR72104, MNHN Paris

**10h50-11h10** Impacts des pollutions urbaines et agricoles sur la prolifération des moustiques par Fréderic Darriet, entomologiste médical, IRD Délégation Occitanie

**11h10-11h30** Chlordécone aux Antilles: un « Tchernobyl » chimique sous les tropiques. Luc Multigner, médecin épidémiologiste, INSERM, Institut de recherche en santé, environnement et travail (Rennes et Pointe à Pitre)

**11h30-12h15** Discussion

**12h15-13h** Pause

**13h Pollutions minières**

**13h-13h20** Les activités minières: un exemple de la complexité d'agir en santé publique environnementale. Michèle Legeas, EHESP Renne

**13h20-13h40** Impacts sanitaires des pollutions minières: approches méthodologiques. Jacques Gardon, médecin épidémiologiste IRD, Hydrosciences Montpellier

**13h40-14h10** Double exposition au Pb et au Hg des populations amérindiennes de Guyane: sur la trace isotopique des sources. Laurence Maurice, IRD, hydrogéochimiste, Laboratoire géosciences environnement de Toulouse

**14h10-14h40** Discussion

**14h40-14h50** Pause

**14h50 Pollutions pétrolières**

**14h50-15h10** Entre culture du risque et culture d'urgence face à la contamination environnementale: le cas des activités pétrolières en Equateur. Sylvia Becerra, sociologue de l'environnement et des risques, Laboratoire Géosciences Environnement Toulouse

**15 h10-15 h20** discussion

**15 h20-16 h Bilan de la journée et table ronde finale**

**16 h Clôture. Jean Jannin, Président de la SFMTSI**

