# Supplementary material for: Pollutions Environnementales et Santé sous les Tropiques
Source: Med Trop Sante Int. 2021 Apr 20;1(2):mtsibulletin.n1.2021.92. [Article in French] doi: 10.48327/mtsibulletin.n1.2021.92 (PMC9022767; doi:10.48327/mtsibulletin.n1.2021.92)
Supplement: Related texts

# Research article: Émissions Anthropiques, Qualité De L'air Urbain ET Impact Sanitaire en Afrique Sub-Saharienne

**Published:** 2021-04-20

**Authors:** C. Liousse, T. Doumbia, C. Granier, E. Gardrat, C. Galy-Lacaux, J.F. Léon, L. Roblou, F. Solmon, H. Cachier, J. Adon, V. Yoboué, E. Assamoi, J. Bahino, M. Doumbia, S. Gnamien, S. Keita, E. N'Datchoh Touré, A. Baeza-Squiban, Annesi-Maesano, A. Akpo, J. Djossou, K. Kouamé, M. Ouafo

**Affiliations:** (Laboratoire d'Aérologie, CNRS/UPS, Toulouse, France); (Laboratoire des sciences de la matière, de l'environnement et de l'énergie solaire, Université Félix Houphouet-Boigny, Abidjan, Cote d'Ivoire); (Université Paris Diderot, Unité de biologie fonctionnelle et adaptative-RMCX, CNRS, UMR 8251, Paris, France); (EPAR Department, UMR-S 707 INSERM and UPMC Paris VI, Paris, France); (Laboratoire de physique du rayonnement, Université d'Abomey-Calavi, Abomey-Calavi, Benin); (Institut Pasteur, Abidjan, Cote d'Ivoire); (Université de Douala, Douala, Cameroun)

Dans un contexte d'urbanisation galopante, l'Afrique sub-saharienne est en proie à une augmentation peu régulée de ses sources anthropiques d'émissions polluantes et par conséquent à une dégradation préoccupante de la qualité de l'air de ses villes et de la santé de ses populations. Pourtant, cette thématique est très peu étudiée dans cette région du monde. Ce n'est que vers les années 2005 que sont apparus les premiers projets scientifiques portant sur ces questions à différentes échelles spatiales (de l'individu à l'échelle régionale). Les résultats que nous allons présenter ici concernent plus particulièrement l'Afrique de l'Ouest et centrale avec des études sur Bamako (Mali), Dakar (Sénégal), Abidjan (Côte d'Ivoire), Cotonou (Bénin), Ouagadougou (Burkina Faso) et Yaoundé (Cameroun). Ils montrent entre autre: (1) des pollutions provenant d'un mélange de sources anthropiques urbaines (feux domestiques, déchets, trafic, industries…) et de sources transportées comme les poussières désertiques venant du Sahel et du Sahara et les polluants émis par les feux de savane, avec de fortes variations saisonnières; (2) des niveaux de concentration des polluants particulaires en zones urbaines 3 à 10 fois plus importants que les normes de l'organisation mondiale de la santé (OMS); (3) la prédominance du carbone organique et des poussières désertiques avec des variations saisonnières importantes; (4) d'importantes variations spatiales des concentrations particulaires à l'échelle de la ville; (5) l'impact pro-inflammatoire des aérosols est corrélé avec l'aérosol carboné; (6) des impacts sur la santé des populations des polluants particulaires en termes de potentiel inflammatoire, de maladies et de décès à l'échelle régionale liées à des infections respiratoires et cardiovasculaires; ces résultats sont en accord avec les estimations de l'OMS et de Lelieveld et al., (2020) qui estiment à près de 1 million par an, le nombre de morts annuel attribués aux pollutions atmosphériques en Afrique, soit une contribution plus importante que les guerres, le paludisme, le Sida ou le tabagisme; (7) des projections dans le futur montrant une importance croissante de la contribution africaine des émissions anthropiques si aucune mesure de réduction n'est envisagée; (8) l'importance des efforts de mitigation sur la réduction des émissions. Ces résultats montrent enfin le type d'études long-terme à mettre en place afin de réduire les incertitudes pesant sur l'estimation de l'impact des émissions anthropiques sur la qualité de l'air et la santé des populations dans cette région du monde.

# Research article: Affections broncho-pulmonaires et pollutions urbaines

**Published:** 2021-04-20

**Authors:** E. Pichard

**Affiliations:** Institut Pasteur, APHP, SFMTSI, Hôpital Pitié-Salpêtrière - Pavillon Laveran, 47-83 Boulevard de l'Hôpital, 75651 Paris cedex 13

S'il parait intuitivement et statistiquement évident que la pollution de l'air dans les milieux urbains des pays pauvres du globe entraîne une augmentation ou une aggravation des pathologies respiratoires, l'analyse de la littérature scientifique apporte de nombreuses interrogations sur ce postulat. Les effets de la pollution intra domiciliaire sont surtout évalués, en particulier chez des enfants avec comme indicateur principal les pneumopathies aiguës. Mais les types de polluants incriminés sont variés, leur mesure est rarement standardisée, la définition même des pneumonies est imprécise, les multiples cofacteurs microbiologiques, climatiques, comportementaux et sociaux sont peu abordés. L'interaction des pollutions intra et extra domiciliaires n'est que probable. La responsabilité de la pollution au cours de l'asthme suscite les mêmes interrogations d'autant que la définition même de l'asthme est peu précise. Chez les adultes, le poids de la pollution pèse surtout sur les pathologies chroniques comme la broncho-pneumopathies obstructives. La responsabilité de la pollution sur les cancers des voies respiratoires chez les non-fumeurs est probable mais intriquée avec celle des cofacteurs génétiques et épigénétiques. La physiopathologie et les conséquences cellulaires et moléculaires des polluants au cours des atteintes respiratoires dans les pays tropicaux ne sont souvent qu'une extrapolation et une compilation de données recueillies dans le contexte environnemental des pays développés. La diversité des cofacteurs artisanaux, industriels, agricoles, miniers ou climatiques aigus est à prendre en compte pour proposer des mesures préventives. Seules des pollutions aiguës comme celle de Bhopal ont pu clairement alerter sur la mortalité directe de la pollution par atteinte respiratoire.

Ces incertitudes seront partiellement levées par la surveillance des indicateurs de maladies respiratoires au fil de la réduction souhaitée ou de l'aggravation redoutée de la pollution dans les milieux urbains en expansion dans les pays dits tropicaux. Globalement la pollution est devenue une des contraintes de la transition épidémiologique dans ces pays, se traduisant par une augmentation du poids des pathologies broncho-pulmonaires chroniques et la réduction de celui des pathologies infectieuses.

# Research article: Les Chimpanzés Sauvages, Victimes ET Sentinelles Des Pollutions Environnementales?

**Published:** 2021-04-20

**Authors:** S. Krief

**Affiliations:** (vétérinaire, primatologue, UMR 7206, MNHN/CNRS/Paris Diderot, Sebitoli Chimpanzee Project Ouganda), P. Spirhanzlova (Sebitoli Chimpanzee Project), J.B. Fini (MNHN)

Dans le Nord du parc national de Kibale, à l'Ouest de l'Ouganda, les chimpanzés (*Pan troglodytes schweinfurthii*) et les babouins (*Papio anubis*) consomment régulièrement le maïs des champs qui entourent l'aire protégée et se nourrissent le long de la route bitumée qui traverse la zone. Un quart des 66 chimpanzés étudiés sur le territoire de Sebitoli (25 km^2^) présentent des malformations congénitales faciales et des membres ou des troubles de la reproduction. De plus, 30 babouins de cette même zone ont également de sévères malformations nasales. Nos enquêtes auprès des fermiers et des sociétés de thé voisines révèlent l'usage fréquent de pesticides. Les analyses de sol, de sédiments et d'eau de la zone montrent que certains excèdent les limites recommandées (DDT et *pp'*-DDE, chlorpyrifos). L'imidaclopride qui enrobe les semences de maïs utilisées est trouvé dans la chair des poissons dans des zones éloignées de plusieurs kilomètres des champs. Des échantillonnages passifs dans les rivières traversant le territoire de Sebitoli montrent que parmi les 13 pesticides trouvés en cocktail, le carbofuran, le DEET, 2.4-D amine, sont présents en grande quantité. Les échantillons d'eau collectés sur ces sites ont des activités de perturbations de l'axe thyroïdien in vivo et produisent des malformations et des troubles comportementaux sur les têtards de *Xenopus laevis*.

Le long de la route, les concentrations atmosphériques de O_3_, NO_2_, SO_2_ de BTEX n'excèdent pas les limites recommandées, mais plus de 5000 bouteilles plastiques ont été collectées par nos équipes le long des 4 km de la route en quelques mois. Pour la première fois, nous avons détecté la présence de bisphénol A et S dans les poils des chimpanzés.

Compte-tenu des polluants mis en évidence dans cette zone et de leurs effets de perturbation endocrine, notre hypothèse est qu'ils puissent contribuer à expliquer les malformations et troubles de la reproduction observés chez les primates non humains. Menacés de disparition dans un futur proche, les chimpanzés sont également des sentinelles des risques sanitaires auxquels les humains sont exposés.

# Research article: Impacts Des Pollutions Urbaines ET Agricoles Sur la Prolifération Des Moustiques

**Published:** 2021-04-20

**Authors:** F. Darriet

**Affiliations:** entomologiste médical IRD, délégation Occitanie

Scientifiques et politiques s'accordent à dire que notre planète subit actuellement une sixième extinction du vivant. Alors que la diversité biologique des milieux terrestres et aquatiques s'érode toujours plus chaque jour, les organismes nuisibles à la santé des hommes, des animaux et des plantes n'ont par contre jamais été aussi nombreux. Les grandes villes se sont multipliées sur tous les continents en générant des pollutions domestiques et industrielles, et, dans les campagnes, l'utilisation souvent irraisonnée des intrants agricoles (engrais et pesticides) déciment les plantes et les animaux. Cent quatre-vingt millions de tonnes d'engrais et 2,4 millions de tonnes de pesticides sont déversés chaque année dans le monde. Ces pollutions, qu'elles soient urbaines ou rurales, ont un impact considérable sur la biologie des moustiques. Aujourd'hui, les espaces urbains sont devenus de véritables nids à moustiques et dans les campagnes, l'usage combiné des engrais et des pesticides favorise leur pullulation. L'ironie de cette histoire moderne du monde est que les différents facteurs qui favorisent la pullulation des ces insectes vecteurs de pathogènes à l'Homme sont ceux là mêmes qui déciment une bonne partie de la biodiversité.

# Research article: Chlordécone Aux Antilles: Un « Tchernobyl » Chimique Sous Les Tropiques

**Published:** 2021-04-20

**Authors:** L. Multigner

**Affiliations:** INSERM, Institut de recherche en santé, environnement et travail (Rennes et Pointe à Pitre)

L'usage du chlordécone aux Antilles, alors même que sa persistance environnementale et sa dangerosité étaient parfaitement établies, a conduit à la pollution d'au moins un tiers des surfaces agricoles et autant du littoral marin. Il en résulte une contamination de la chaîne alimentaire locale, végétale et animale, atteignant l'ensemble de la population.

Au cours de mon intervention je retracerai les circonstances qui ont conduit à l'usage de cet insecticide, son profil toxicologique, les risques pour la santé de la population ainsi que les conséquences sociales et économiques d'une pollution dont la durée est estimée à plusieurs siècles.

# Research article: Les Activités Minières: Un Exemple de la Complexité D'agir En Santé Publique Environnementale

**Published:** 2021-04-20

**Authors:** M. Legeas

**Affiliations:** EHESP Rennes

Agir en santé publique environnementale, surtout « sous les tropiques » pose de nombreuses questions, de plusieurs natures: la dégradation des écosystèmes et la perte de biodiversité, la prise en compte des besoins des populations en matière économique et sociale, la création de situations induisant un vaste registre de problèmes de santé pour ces populations, les inégalités dans toutes leurs dimensions et des questions éthiques au regard de nos modèles de société.

L'exposé visera à développer ces différentes questions, à travers deux situations réelles: les mines d'or de la région de Kayes au Mali et les mines de phosphate dans le gouvernorat de Gafsa en Tunisie.

# Research article: Impacts Sanitaires Des Pollutions Minières: Approches Méthodologiques

**Published:** 2021-04-20

**Authors:** J. Gardon

**Affiliations:** médecin épidémiologiste IRD, Hydrosciences Montpellier

La pollution est aujourd'hui la première cause de mortalité au monde. L'industrialisation de nos sociétés génère d'innombrables sources de contaminations de l'air, de l'eau et des sols, qui entraînent le décès prématuré de plus de neuf millions de personnes chaque année. Avec plus six millions de décès attribuables, c'est la pollution de l'air qui constitue la principale préoccupation, notamment dans les pays du sud. La pollution chimique des sols est un enjeu important à long terme, provoquant actuellement un demi-million de décès par an. La pollution de l'eau est avant tout microbiologique, elle est responsable d'environ deux millions de décès, avec un très fort impact chez les enfants. Elle régresse globalement avec l'amélioration de conditions de vie, l'assainissement et la potabilisation des eaux utilisées pour l'alimentation humaine. Pourtant, au moment où la qualité microbiologique s'améliore, l'eau est de plus en plus souvent contaminée par des composés chimiques, organiques ou inorganiques, dont l'impact est encore mal évalué.

La transition énergétique demande toujours plus de matières premières, très souvent extraite avec peu de contraintes environnementales dans les pays en voie de développement. Dans le même temps, les villes et les littoraux fortement anthropisés cumulent des pollutions complexes.

Les activités minières indispensable aux développements technologiques sont parmi les plus polluantes, contaminant l'environnement avec de nombreux éléments inorganiques, dont certains sont très toxiques. L'arsenic, le plomb, le mercure et le cadmium ont des toxicités bien établies, provoquant des cancers, des maladies dégénératives ou des retards de développement chez les enfants. Par ailleurs, certains éléments inorganiques, qui sont essentiels à la santé en faible quantité, sont toxiques à concentrations élevées, comme par exemple le manganèse.

En utilisant des exemples concrets, issus de la littérature et de travaux personnels en Amérique Latine et en Afrique, les situations où des pollutions inorganiques peuvent avoir des conséquences sanitaires seront décrites en commentant les approches méthodologiques.

# Research article: Double Exposition AU Pb ET AU Hg Des Populations Amérindiennes de Guyane: Sur la Trace Isotopique Des Sources

**Published:** 2021-04-20

**Authors:** L. Maurice, F. Barraza, S. Goix, I. Blondet, L. Laffont, J. Sonke, M. Ho-A-Chuck, J. Tablon, P. Brousse, E. Schreck

En Guyane française, des niveaux élevés d'exposition au mercure (Hg) ont été relevés en particulier chez les populations amérindiennes vivant le long de l'Oyapock. Le mercure est un puissant neurotoxique qui traverse la barrière placentaire et dont les conséquences sur le développement des enfants peuvent entraîner des effets irréversibles. D'une étude menée entre 2012 et 2015 dans les populations Wayãpis et Teko riveraines du fleuve Oyapock et de la rivière Camopi, il a été observé que la distribution des concentrations en mercure dans les cheveux est inversée par rapport à celle mesurée dans les sédiments et les poissons de ces mêmes sites, plus contaminés au niveau des zones orpaillées (dans le bassin de la Camopi). En effet, le niveau d'imprégnation de ces populations est significativement plus élevé dans les communautés de Trois Sauts (6,314 ± 2,108 μg Hg.g-1), en amont, que dans celles vivant dans la région d'orpaillage (4,064 ± 2,262 μg Hg.g-1). À Camopi, 29% des personnes étudiées (N = 60) dépassent la valeur de 5 μg.g-1 (valeur limite recommandée par l'OMS) contre 65% à Trois Sauts (N = 51).

Parallèlement, dans ces mêmes communautés, des cas de saturnisme ont été détectés par l'ARS et une étude a été conduite au sein de 15 familles dont tous les enfants présentent des niveaux de plomb (Pb) dans le sang élevés (de 5,7 à 35 μg.dL-1) supérieurs à 5 μg.dL-1 (valeur seuil OMS, 2018). L'intoxication chronique par le plomb peut conduire à divers troubles essentiellement liés à son accumulation dans le système nerveux central, source d'altérations des fonctions cognitives chez les jeunes enfants et dans le rein Elle peut aussi induire des troubles du système cardiovasculaire et du développement embryonnaire.

Les analyses des isotopes stables de Hg et de Pb réalisées dans les cheveux et le sang de ces communautés, comparées à celles des sources potentielles de ces métaux toxiques, ont permis de mettre en évidence le rôle majeur des habitudes alimentaires dans ces niveaux d'exposition.

Les populations de Trois Sauts, isolées, consomment plus de poissons pêchés localement que celles de Camopi, qui ont une alimentation plus variée grâce à la présence de petits commerces. Les cheveux des femmes portent la signature isotopique des poissons de rivière de leur lieu de vie (enrichissement de ~+2 ‰ pour δ^202^Hg sans changement de Δ^201^Hg). Au contraire, les cheveux des hommes montrent une signature isotopique issue d'un mélange de poisson local et de poisson marin, suggérant, lors de leurs déplacements, la consommation de poissons marins (en conserve).

Le couplage des analyses de plomb total et des fractionnements isotopiques de cet élément dans les sols, les tubercules de manioc, les aliments (bols alimentaires, gibier) et les plombs de chasse, ont permis, en les comparant aux signatures mesurées dans le sang des 15 enfants étudiés, de tracer les sources de cet élément toxique. Les résultats obtenus mettent en évidence l'importance, dans les niveaux de plombémie mesurés dans les populations locales de Trois Sauts, de la consommation quotidienne de manioc (tant sous forme d'aliments solides que liquides) comme celle de l'usage et la manipulation de plombs ou de consommation de viande de gibier près des impacts des plombs de chasse.

Les communautés amérindiennes vivant le long du fleuve Oyapock, et plus particulièrement celles installées dans les régions les plus reculées, en amont sont donc exposées, via leurs habitudes alimentaires, à deux métaux neurotoxiques. Cependant, intervenir sur ces habitudes mettrait en péril à la fois leur équilibre nutritionnel et leur cohésion culturelle. Des études sont aujourd'hui prévues pour identifier les variétés de manioc qui présentent un facteur de bioaccumulation faible et des campagnes d'information sur les espèces de poissons recommandées pour les femmes enceintes et les jeunes enfants ainsi qu'un biomonitoring sont en place depuis plusieurs années.

# Research article: Entre Culture du Risque ET Culture D'urgence Face à La Contamination Environnementale: Le Cas Des Activités PétrolièRes en Équateur

**Published:** 2021-04-20

**Authors:** S. Becerra

**Affiliations:** Laboratoire Géosciences Environnement Toulouse

Le pétrole est le « *sang de la terre* » et la principale source d'énergie fossile au monde. L'Amazonie équatorienne, terre d'exploitation pétrolière et de colonisation agricole, est un symbole des richesses naturelles de la planète, mais aussi le témoin des dommages et risques sanitaires environnementaux causés par les activités humaines (Becerra, Maurice, Desprats-Bologna, 2018).

Si certaines pratiques industrielles ainsi que de nouvelles réglementations ont permis de réparer partie des impacts sociaux (réglementation et politique de compensations sociales) et environnementaux (politiques de remédiation des passifs environnementaux et relogement des personnes exposées), l'expérience sociale de la contamination de l'environnement est relativement stable: « *tout est contaminé* »; « *on est tous concernés* »; « *les entreprises nous mentent* », etc. Au–delà du constat, la question qui se pose est celle du rapport au risque: qu'est-ce qui est défini comme risqué localement?

Loin de la définition consentie de la culture du risque dans les pays occidentaux, nos résultats montrent que la stratégie adoptée par les habitants confrontés aux pollutions pétrolières en Amazonie équatorienne n'est pas toujours de réduire leur exposition sanitaire, car d'autres risques sont en jeu. La contamination est ainsi parfois instrumentalisée. Conservée comme preuve des dommages pétroliers, elle change alors de statut: elle passe paradoxalement de menace pour la santé à « *arme pour défendre ses droits* » (pour reprendre les termes d'un habitant). Elle devient notamment une sorte de « *monnaie d'échange* » pour obtenir une compensation monétaire ou matérielle (biens d'usage immédiat; emplois).
